# Influence of Irradiation Parameters on Structure and Properties of Oak Wood Surface Engraved with a CO_2_ Laser

**DOI:** 10.3390/ma15238384

**Published:** 2022-11-25

**Authors:** Jozef Kúdela, Ivan Kubovský, Michal Andrejko

**Affiliations:** Faculty of Wood Sciences and Technology, Technical University in Zvolen, T.G. Masaryka 24, 96001 Zvolen, Slovakia

**Keywords:** CO_2_ laser, engraving, oak wood, irradiation dose, morphology, chemical composition, colour, wetting

## Abstract

The work investigates the effects of CO_2_ laser parameters (laser power and raster density) on wood mass loss in oak wood and impacts on its morphology, chemical structure, and surface properties (colour and hydrophilicity). The energy amount supplied onto the wood surface with a laser beam under different combinations of the irradiation parameters was expressed through a single variable—total irradiation dose. The mass loss was confirmed as linear-dependent on the irradiation dose. With the mass reduction, the roughness was enhanced. The roughness parameters *Ra* and *Rz* increased linearly with the mass loss associated with the increasing irradiation dose. The FTIR (Fourier transform infrared spectroscopy) spectroscopy also detected chemical changes in the main wood components, influencing primarily the wood colour space. Conspicuous discolouration of the engraved wood surface was observed, occurring just at the minimum laser power and raster density. The additional increasing of laser parameters caused a novel colour compared to the original one. The detected dependence of wood discolouration on the total irradiation dose enables us to perform targeted discolouration of the oak wood. The engraved surfaces manifested significantly better wettability with standard liquids, both polar and non-polar, and higher surface energy values. This guarantees appropriate adhesion of film-forming materials to wood. Identification of the changes in wood surface structure and properties, induced by specific CO_2_ laser-treatments, is important for obtaining targeted discolouration of the wood surface as well as for the gluing or finishing of the surfaces treated in this way.

## 1. Introduction

The technology of material treatment using laser irradiation has met a wide range of applications in cutting and surface treatment of metallic and non-metallic materials, including wood and wood-based ones [1,2,3,4,5,6,7,8,9]. At present, considerable attention is devoted to studying the performance of surfaces engraved with CO_2_ lasers, aiming for purpose-oriented changes to wood structure and properties [6,8,10,11,12,13,14,15,16,17,18,19,20].

The energy concentrated in a laser beam and supplied onto a specific spot on the engraved wood surface is converted to heat. This heat causes thermal degradation of wood surface structures in the area of the heat-affected zone. The high energy values cause the wood surface layer to sublimate, which is accompanied by the formation of a carbonized or melted layer. The wood surface treated in this way exhibits changes in its chemistry and structure, reflected subsequently in the changes in morphology, colour, and wetting performance with liquids [18,21,22,23,24]. This energy can be controlled by the laser power, the rate of laser head movement, focal distance, and raster density [11,12,16,22,25,26,27,28]. Apart from the energy supplied amount and its concentration, the thickness of the heat-affected zone depends considerably on the wood species concerned [11,12,19,22,23].

The results of FTIR spectroscopy and gas chromatography showed [21] that wood irradiation with a CO_2_ laser caused a decrease in the amount of polysaccharides depending on the energy amount supplied. The degradation primarily concerned hemicelluloses and a part of the amorphous share of cellulose. There were also evident changes in the lignin structure. Furthermore, in the case of chemical changes, the species-related change has been recognised as significant [21,22,23,29].

Chemical changes in the wood structure also result in its colour changes. These changes are caused by the bonds’ cleavage in the chromophore structures that are responsible for the colour of the wood [10,11,21,24,30,31,32]. Kúdela et al. [11] document colour changes just at the minimum laser power and raster density values. By increasing the laser power and raster density, the total colour difference values in all cases were much beyond the value of 12, representing a totally new colour compared to the original one [33]. Higher raster densities caused more pronounced wood mass reduction at the overlapped spot and a more evident discolouration [8,18,25,34]. The quantification of the energy amount supplied on the wood surface with a laser beam and the control of the total irradiation dose may provide conditions for targeted changes to the colour space for the relevant wood species treated with a CO_2_ laser.

The microscopic observations indicate [22,23] that wood surface treatment with a laser beam may reduce the wood surface roughness by melting the cells down to a depth of several micrometres, but without carbonisation. The work [10] reports significant morphological changes manifested through more pronounced roughness associated only with higher irradiation values, mainly due to the carbonisation of the wood surface layer. The opposite effect (more roughness), however, may occur when engraving the wood surface with a laser [11,12,27]. The paper [12] reports that for engraving beech wood surface with a CO_2_ laser, at low laser power values (4%), there was a moderate decrease in the roughness parameters along the fibre direction (compared with the sanded surface of the referential samples). The roughness parameter values were lower than in the sanded referential specimens. The roughness was reduced by the influence of the laser beam causing the ablation of the cell elements released by sanding, and the same has been confirmed by [19]. Through increasing the laser power and density, an essential increase in roughness was observed [11,12]. For engraved wood surfaces, an important influence on the roughness parameters for all the laser parameters (laser power, laser head movement rate, and raster density) was confirmed, for wood species as well as the anatomical direction [9,11,12,16,18,27]. 

Wood surface treatment with a laser beam also impacts wood surface wetting with various liquids [10,22]. The contact angle values are a significant indicator for predicting the adhesion of glues and coating materials to such surfaces. Contact angle values are also the background for determining the thermodynamical characteristics of the wood surface—surface free energy and its components [35,36,37,38,39]. 

Haller et al. [22] report that the pine wood surface melted but not yet carbonised due to treatment with a CO_2_ laser (supposed temperature up to 200 °C) manifested worse wetting with water compared to the non-modified surface. The melt layer reaching down to several micrometres enhanced the surface hydrophobicity to a considerable extent. This was subsequently reflected in contact angle values higher than 90° and in decelerated drop soaking into the wood. The results obtained by [23] indicate that the irradiated surface did not show lower wetting; the contrary was more common. The last cited paper implies that the surface energy of wood treated with the laser was kept without significant changes. The total surface energy was low, with a dominant disperse component. In this case, the laser radiation parameters were different from the ones used by Haller et al. [22]. The equivocal effect of increasing the radiation dose on beech wood wetting with standard liquids has not been confirmed either by [10]. 

The analysis of the whole summary of the published results shows that the energy absorbed into the surface during CO_2_ laser treatment induces chemical changes in the main components of wood, in its morphology, colour, and hydrophilicity or hydrophobicity of the wood surface. The differences between the authors can be explained by using lasers with different technical parameters based on the different methodical approaches. Despite this fact, the results clearly show that the appropriate adjustment of CO_2_ laser radiation parameters can be a means for targeted wood surface modification, in accordance with the current requirements. For this purpose, it is necessary to study the whole range of changes in wood surface properties, using the same methods. 

The aim of this work was to evaluate comprehensively the influence of infrared laser radiation generated with a CO_2_ laser on the surface structure and properties of oak wood. This aim comprehended the following partial tasks:

- to determine the total amount of energy supplied with a laser beam onto the wood surface under varying CO_2_ laser parameters (laser power, raster density);
- to assess the impact of the total radiation dose on the wood mass loss;
- to assess the impact of the specific method of surface treatment on the wood surface chemical structure and morphology quantified through roughness parameters;
- to inspect colour changes depending on different laser parameters and to identify how the total energy supplied influenced the changes in the colour coordinates;
- to find out the influence of engraving on the wetting process and on the surface free energy.

## 2. Materials and Methods

### 2.1. Experimental Material and Irradiation Parameters

**Figure 1:**
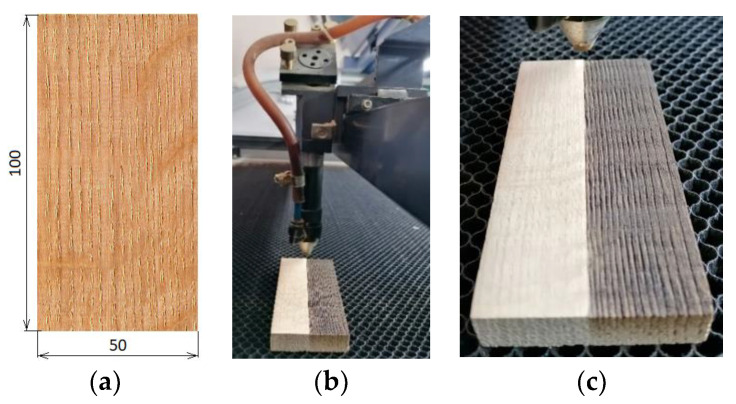
Engraving oak wood surface with a CO_2_ laser CM-1309. (**a**) radial surface of a test specimen, (**b**) engraving process, (**c**) engraved surface).

The experiments were carried out on specimens prepared from ring-porous oak wood. The specimen dimensions were 50 mm × 100 mm × 15 mm (width × length × thickness). Prior to the grinding, the surfaces were sanded with a sandpaper with a grain size of P180. The irradiated (engraved) surfaces were radial, sized 50 mm × 100 mm (Figure 1a).

During the engraving, the specimens were placed under the focus of a CO_2_ focusing lens of laser equipment CM-1309 (Shenzhen Reliable Laser Tech, Shenzhen, China). The specimen distance from the lens was 17 mm. The laser head moved over the specimen surface parallel to the grain, at a constant speed of 350 mm·s^−1^ (Figure 1b,c). The radiation energy varied with varying laser power and raster density (number of paths per one millimetre of width). Three specimen sets were prepared. The first was engraved at a laser power of 8% (derived based on the maximal laser power, representing the resonator output value of 137.5 W), the second at 12% laser power, and the third at 16% power. The laser power was measured with measuring equipment FieldMaxII-TOP (Coherent, Wilsonville, OR, USA). The equipment sensor PM150-50C placed at the level of the specimen surface measured the power of laser beams perpendicular to the surface.

The numbers of paths per one millimetre of the width were 2, 5, 10, 20, and 30 (defined as density values in mm^−1^), representing altogether 5 combinations, each consisting of four specimens, plus four referential ones (0 mm^−1^). Under the experimental conditions, all the specimens were irradiated uniformly over their length and width (Figure 1). The laser parameters (laser power, head movement rate, focal distance and raster density) were decided based on our experience with previous measurements (Kúdela et al. 2019, 2020, 2021) and on the knowledge collected from the literature referred to in the Introduction.

The amount of the energy supplied was expressed as the amount of irradiation dose *H*’. The total irradiation dose per unit area of irradiated specimens was determined in accordance with Kubovský et al. [32]. The authors calculated the irradiation dose for one route of the laser beam according to their equation:

(1) $$H=\frac{P_{e}\cdot \tau }{A}=\frac{P_{e}\cdot x}{A\cdot v}$$

where *P*_e_ is the effective laser power on the specimen surface, *τ* is irradiation time during one path (ratio between the specimen dimension *x* and speed *v*), and *A* is area irradiated at one laser beam path.

The irradiation dose *H*’ related to a unit area of the irradiated specimen was subsequently calculated according to the equation:

(2) $$H\prime =H\cdot n\cdot y=\frac{P_{e}\cdot x\cdot n\cdot y}{A\cdot v}$$

where *n* is the raster density, and *y* is the width of the track left by the laser beam after one path on the wood surface. The track width was measured with the aid of a camera Leica EC3 (Leica Microsystems, Heerbrugg, Switzerland).

### 2.2. Determining the Weight Ratio of the Wood Mass Loss

For determining the ratio of wood mass loss after engraving the wood surface with a CO_2_ laser, new specimens were prepared from the same experimental material. The specimens were dried out to a zero-moisture content at a temperature of 103 ± 2 °C. The dry specimens were removed from the drying chamber and placed into an exicator with silica gel. After the cooling, the specimens were weighed on a laboratory scale with a precision of 0.001 g (mass *m*_01_). The dried and weighed specimens were engraved at specified laser powers and raster densities. After this process, the specimens were weighed again, the result of which was the mass *m*_02_. Then, the equation

(3) $$\Delta m=\frac{m_{01-}m_{02}}{m_{01}}\cdot 100$$

was used for calculating the weight ration for the wooden mass loss. 

### 2.3. Evaluation of Surface Morphology

Wood surface morphology after the laser treatment was evaluated from the viewpoints of anatomy and physics. The roughness profiles were measured with a profilometer Surfcom 130A (Carl Zeiss, Oberkochen, Germany) consisting of a measuring unit and an evaluation unit. The changes to the wood surface morphology induced by CO_2_ laser engraving were evaluated through the following roughness parameters: *R*a (mean arithmetic deviation), *R*z (the maximum peak height plus the maximum depression depth within the cut-off, or sampling length), *R*t (the maximum peak height plus the maximum depression depth within the entire evaluation length) and *RS*m (mean distance between the trenches—arithmetic mean calculated from distances between the profile unevenness within the sampling length).

The roughness was measured on the irradiated radial surfaces, parallel with and perpendicular to the grain. The roughness evaluation started by filtering off the waviness from the basic profile, and then the roughness curve was transferred onto the basic line. The entire transverse length consisted of run-up segment *l*_r_, five sampling length (cutoff) segments *λ_c_*, and the over travel segment; *l*_p_. The basic lines were chosen from the interval 0.025–8 mm, based on the preliminary measured roughness parameters *R*a and *R*z. For studying the structure of the engraved oak wood surfaces, a light microscope Leica MZ 9.5, camera Leica EC3 was used, and a digital microscope Keyence VHX 7000 (Keyence International, Mechelen, Belgium). Microscopic slides of the transverse cuts were prepared following the methods described in [12].

### 2.4. Detecting Chemical Changes after Engraving

Chemical changes were inspected in specimens irradiated at all the tree laser power and raster density values 10, 20, and 30 mm^−1^. For this purpose, FTIR Analysis (Fourier Transform-Infrared Spectroscopy) was used. FTIR spectra of the engraved wood surfaces were recorded on a Nicolet iS10 FTIR spectrometer (Thermo Fisher Scientific, Waltham, MA, USA), equipped with Smart iTR using an attenuated total reflectance (ATR) sampling accessory attached to a diamond crystal. The spectra were acquired by accumulating 64 scans at a spectral resolution of 4 cm^−1^ in an absorbance mode from 4000 to 650 cm^−1^ standardised using the baseline method. The obtained results were analysed using OMNIC 9.0 software (Thermo Fisher Scientific, Waltham, MA, USA). Measurements were performed on four replicates per a sample. Each specimen was measured on the radial surface.

### 2.5. Colour Measurement

The colorimetric values L*, a* and b* on the referential and engraved specimens were measured with a spectrophotometer Spectro—guide 45/0 gloss (BYK-GARDNER GmbH, Geretsried, Germany). The measurements were taken at ten spots per one specimen. The colour differences ΔL*, Δa*, Δb* under different irradiation conditions and the total colour difference Δ*E* were determined according to the following equations:

(4) $$\Delta L^{*}=L_{2}-L_{1}$$

(5) $$\Delta a^{*}=a_{2}-a_{1}$$

(6) $$\Delta b^{*}=b_{2}-b_{1}$$

(7) $$\Delta E=\sqrt{\Delta L^{*}{}^{2}+\Delta a^{*}{}^{2}+\Delta b^{*}{}^{2}}$$

where the index “1” represents the colour coordinate value in the referential specimen (not engraved) and the index “2” indicates the value of the colour coordinate of the wood specimen irradiated with the CO_2_ laser.

### 2.6. Wood Surface Wetting with Liquids and Determining the Surface Free Energy

Wood wetting with standard liquids was performed, together with measuring the contact angles during the entire wetting process until the complete drop soaking into the substrate were realised with the aid of a goniometer Krüss DSA30 Standard (Krüss, Hamburg, Germany). The wetting process as such was evaluated using a software package DSA3 (Krüss, Hamburg, Germany). Two testing liquids with different polarities were used—redistilled water and diiodomethane. The reasons for using these two liquids follow from [40].

The wood wetting was realised by applying a drop of the relevant liquid with a volume of 0.0018 mL onto the substrate. After the drop contact with the wood surface, a camera scanned the time history of the drop shape in the fibre direction, from the first contact up to the complete soaking. The scanning frequency was adjusted according to the wetting process duration. The drop shape analysis and determining the contact angle were performed by a circle method.

Immediately after applying the drop onto the surface, the contact angle value *θ*_0_ was measured. Based on the parameter *d* (drop width) variation, the moment on the advancing contact angle conversion into the receding one was identified. The contact angle measured at this moment was defined as the “equilibrium” contact angle—*θ*_e_. The contact angle values were measured at twelve different spots on each specimen.

The values of contact angles *θ*_0_ and *θ_e_*, were used, following the methods described in [41], for the calculation of the contact angle corresponding to an ideally smooth surface *θ_w_*. This angle was subsequently used for determining the surface free energy *γ_S_* and its disperse and polar shares *γ_S_^d^* and *γ_S_^p^*. The disperse share was obtained from the wood wetting with diiodomethane, the polar share with water. The total surface free energy was defined as the sum of the polar and non-polar components, according to [40].

## 3. Results and Discussion

The data concerning the wood mass loss, chemical changes, and the changes in the morphology and performance of wood surface engraved at variable laser power and raster density values are presented and analysed in the following sections.

### 3.1. Determining the Total Irradiation Dose

The values *H*´ of the irradiation dose delivered over a unit area were calculated according to Equation (2), with parameters expressing conditions for the relevant CO_2_ laser irradiation. These results are listed in Table 1. The total irradiation dose ranged from 6 to 185 J∙cm^−2^, with the lowest value obtained at the minimum (8%) laser power and the lowest raster density (2 mm^−1^), and the highest at the respective values of 16% and 30 mm^−1^.

**Figure 2:**
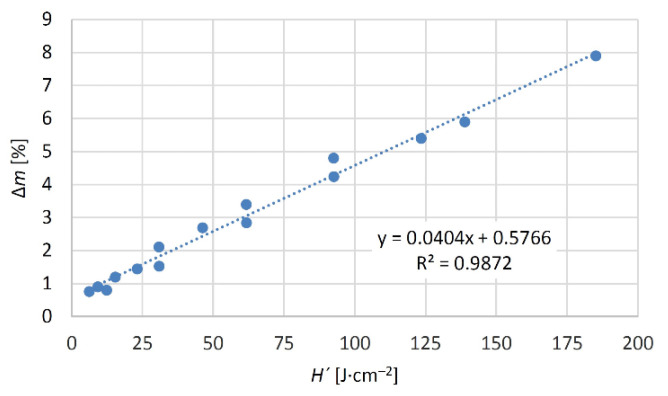
Wood mass loss as a function of irradiation dose.

The increasing irradiation dose was attained by a linear increase in the weight rate of the wood mass loss (Figure 2). Quantitatively similar wood mass loss could also be attained by adjusting the focal distance of the lens and the rate of the laser head movement [16,42]. It was confirmed that all the irradiation parameters impacting s the radiation energy significantly (laser power, head shifting rate, focal distance, and raster density) could be substituted with a single variable—irradiation dose *H*´. Non-uniform mass loss in oak wood was accordingly also reflected in the wood morphology.

### 3.2. Morphology of Wood Surfaces Engraved with CO_2_ Laser

The surface morphology of laser-engraved oak wood was evaluated based on experimentally obtained roughness profiles, parallel with and perpendicular to the grain, in the radial direction. The basic statistical characteristics for the roughness parameters *R*a, *R*z, *R*t, and *RS*m for the relevant raster density range, power values, and the two anatomical directions are in Table 2. The results of a three-way variance analysis confirmed a significant influence of all three evaluated factors (raster density, laser power, and anatomical direction) and their interactions on the evaluated roughness variables.

In the case of parameters *R*a, *R*z, and *RS*m, the number of measurements *n* for each variant was 60. In the case of *R*t, *n* = 12.

The values of all the roughness parameters in the referential specimens were lower than in the engraved specimens, in both anatomical directions, parallel with and perpendicular to the grain. In the referential specimens, significantly higher roughness parameter values were measured perpendicular to the grain, consistently with the orientation of cell elements.

**Figure 3:**
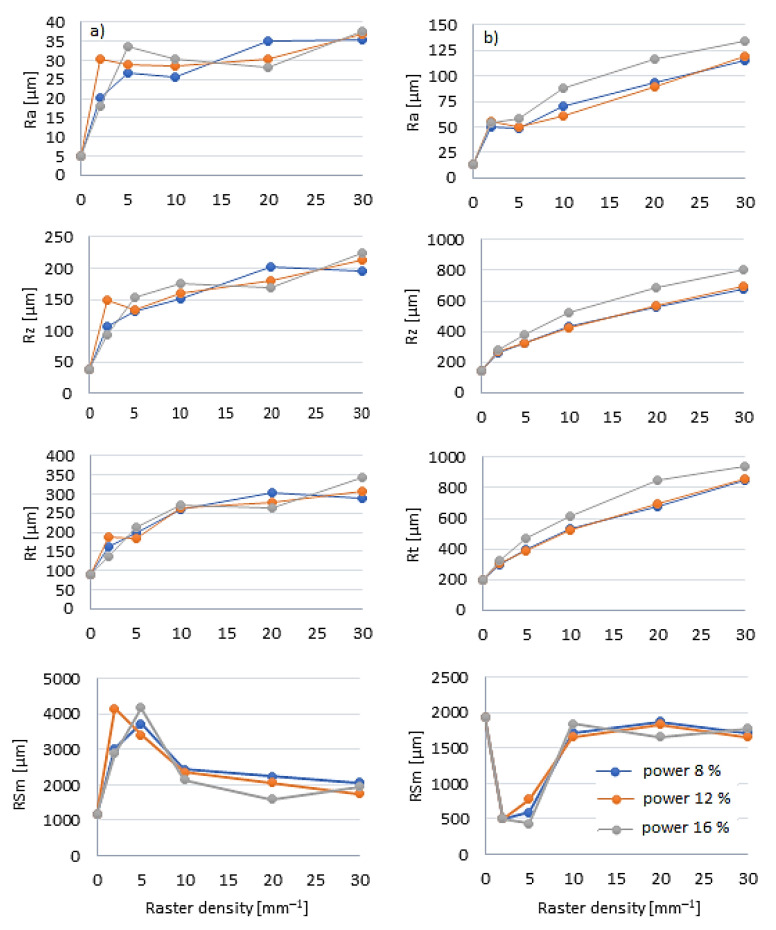
Roughness parameter values *R*a, *R*z, *R*t, and *RS*m in engraved oak wood specimens, dependent on the raster density values, at raster power of 8, 12, and 16%—(**a**) parallel to grain, (**b**) perpendicular to grain.

In the engraved specimens, the roughness parameter values *R*a, *R*z, and *R*t parallel to the grain significantly increased with increasing laser power, and mainly with increasing raster density. At the maximum laser power and the maximum raster density, the roughness parameter increase was several-fold. The roughness parameter values *RS*m, parallel to the grain, increased with increasing raster density as far as 5 mm^−1^; there followed a moderate decrease (Figure 3).

Perpendicular to the grain, the roughness was significantly higher over the whole engraving range. With increasing raster density, the parameter values *R*a, *R*z, and *R*t increased much more than in the longitudinal direction (Figure 3). At the maximum raster density, the *R*a values were by order higher compared to the referential ones. The parameter *RS*m exhibited the most variability compared to the others. The raster-density-dependent change in parameter *RS*m perpendicular to the grain differed from the corresponding change in the parallel direction, in quantity equally as in quality (Figure 3).

Energy concentrated in the laser beam and supplied on a specific spot was converted to heat. The checking of temperature with a thermo-camera, revealed that the wood surface temperature at the moment of contact between wood and beam fluctuated close to the upper measuring threshold of the camera (1000 °C) just at an 8% laser power. Such extreme heat, concentrated on the wood surface within the laser beam with a tiny diameter, caused immediate demolition and sublimation of the thin surface wood layer. Under the same conditions, the thickness of the sublimated layer was not uniform over the irradiated area, due to the wood heterogeneous structure. This was primarily evident in measuring the roughness perpendicular to the grain course. As the direction was radial, the differences observed were a consequence mainly of the differences in the qualitative and quantitative presence of cell elements between the early and late wood.

**Figure 4:**
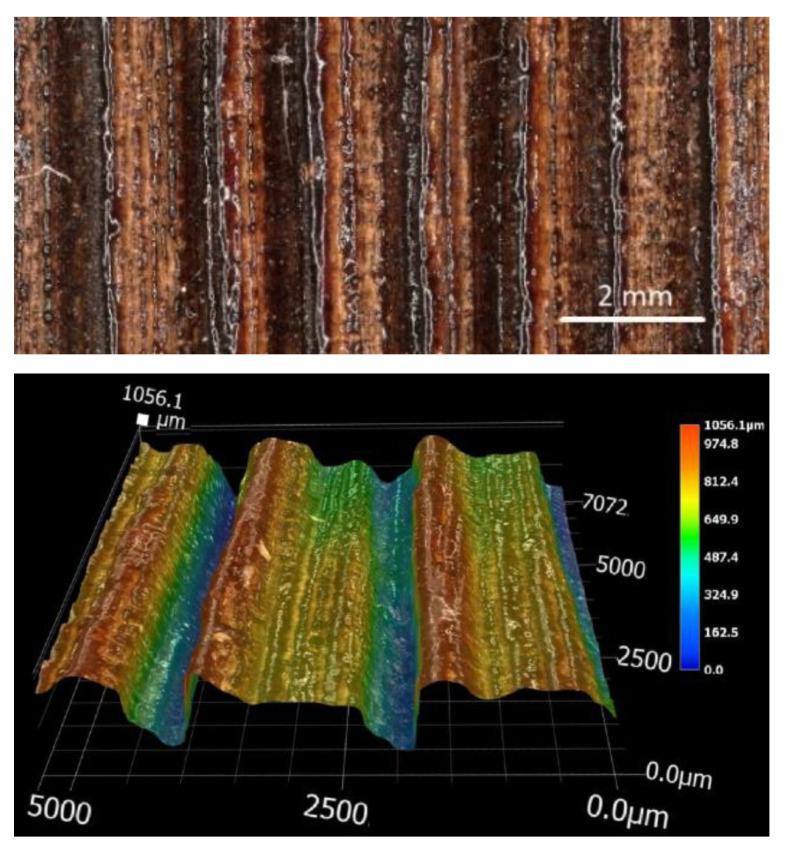
Morphology of oak wood surface engraved with a CO_2_ laser parallel with and perpendicular to grain course, at laser power of 16% and raster density of 30 mm^−1^.

**Figure 5:**
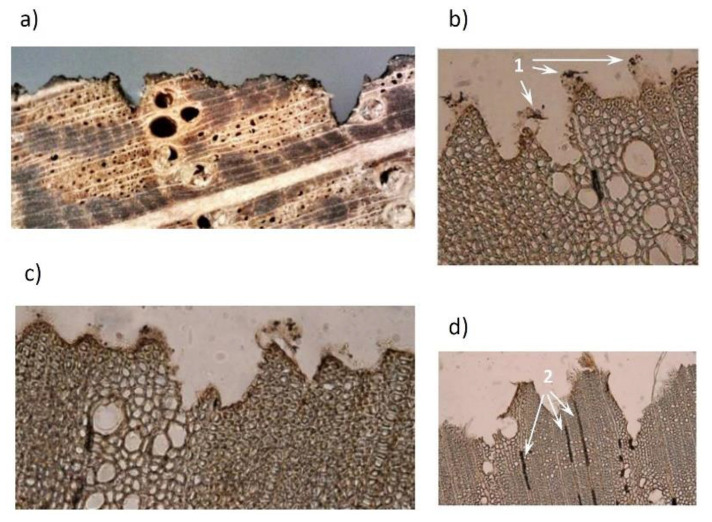
Profile of oak wood engraved with a CO_2_ laser at laser power of 8% and raster density of 20 mm^−1^. (**a**) Transversal cut through oak wood, (**b**–**d**) microscopic slides of transversal cuts, 1—carbonized layer, 2—carbonized parenchymatic cells.

Experimental measurements of roughness and the microscopical observations showed that, apart from the energy amount and concentration, the thickness of the sublimated layer was to a large extent affected by the differences in the structure and properties between the earlywood and the latewood, which was also confirmed for other types of wood [12]. Non-uniform destruction of the wood mass within growth rings, due to the density differences between the early and the late wood, was mainly reflected in more pronounced trenches in the early wood and partly also in distances between the profile unevenness. The altered irradiated wood surface morphology is shown in Figure 4 and Figure 5a.

Oak wood is ring-porous, with early wood vessels having big lumens and thin cell walls, and with libriform fibres exhibiting small lumen diameters and thick cell walls. The roughness profiles measured perpendicular to the fibre course in the radial direction were affected by cell wall degradation, especially in early wood vessels. This resulted in forming deeper depressions in such spots. The paper [12] suggests that the same is true in the case of irradiated tangential surfaces, which is evident on the microscopic slides in Figure 5b–d. If the irradiated oak wood surfaces exhibit dominant tangential shares, the heat supplied onto the surface during engraving penetrates deeper into the wood through its pith rays. The pith rays are composed of parenchyma thin-walled cells that under heat impact degrade easily, up to carbonisation. The carbonised cells in micro-cuts are evidenced as black strips (Figure 5b–d).

**Figure 6:**
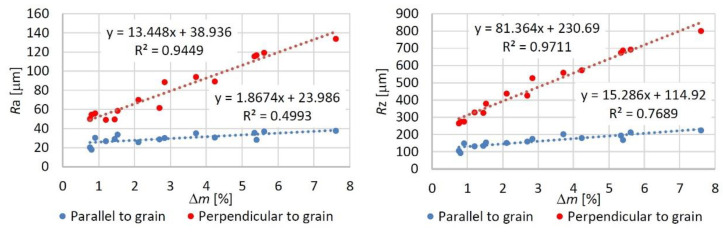
Dependence on roughness parameters *R*a and *R*z on wood mass loss rate.

Non-uniform engraving of the oak wood surface was primarily responded with higher values of parameters *R*a and *R*z, increasing linearly with the wood mass loss rate from the oak wood surface (Figure 6). The results also indicate that the roughness variation was more affected by the raster density than by the laser power, which has also been confirmed by [11,27]. As for the impacts on the wood surface geometry, in this case, all the engraving parameters are possible to substitute with a single variable—the total irradiation dose. With an increasing irradiation dose, the roughness parameters increased linearly. After the engraving, not only the wood surface geometry was changed but also the wood surface chemical structure.

**Figure 7:**
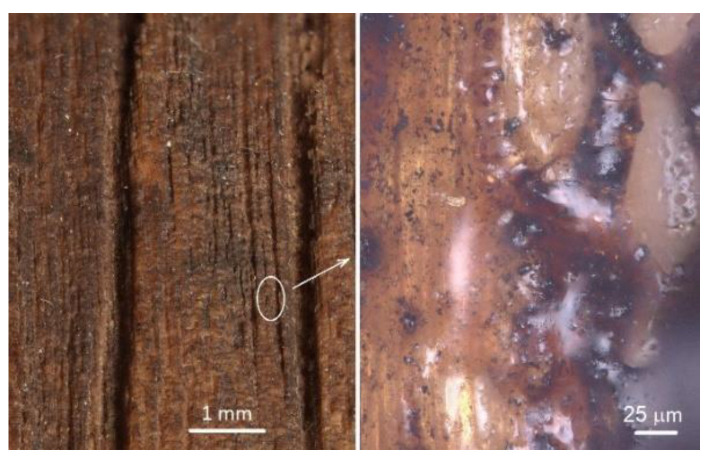
Surface of oak wood engraved with a CO_2_ laser at laser power of 16 % and raster density of 30 mm^−1^. **Left**: radial surface with obvious trenches in early wood. **Right**: melt layer topping the engraved surface—detail.

The engraved wood surfaces, especially those subjected to higher irradiation doses, exhibited a carbonised layer (Figure 4 and Figure 5) characterised by poor stability, and also a melted layer, especially on the late wood surface (Figure 7). These changes were induced mainly by the chemical changes in the surface layers of the studied oak wood.

### 3.3. Changes in the FTIR Spectra

**Figure 8:**
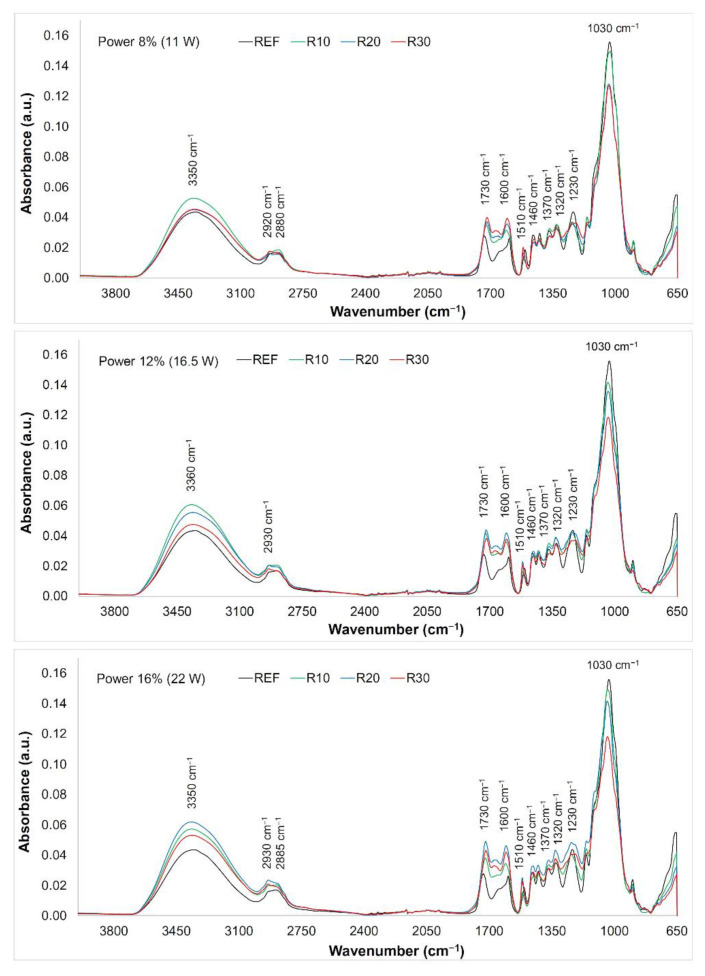
FTIR spectra of oak wood surface engraved with varying power (designations R10, R20, and R30 in the picture represent raster densities of 10, 20, and 30 mm^−1^).

FTIR spectra were measured on the native wood surface and the surface engraved with CO_2_ laser (for used raster densities of 10, 20, and 30 mm^−1^). All the spectra were evaluated altogether, as the differences between them were small. We can see (Figure 8) that the variance in the band from 3100 to 3600 cm^−1^ was minimum. This very broad peak has been assigned to the O−H vibrations in the lignin structure, in cellulose fibres, and in hemicelluloses [43]. The changes in the band absorbance with a peak at 3350–3360 cm^−1^ could be observed at all three laser power values used. Increasing absorbance was especially evident for lower raster density values; conversely, at higher raster densities, the absorbance decreased. This phenomenon may be backed-up with a higher rate of splitting the O−H bonds in the molecules of the water bound to the wood, due to higher amounts of the energy supplied [44]. The interval from 2900 to 2850 cm^−1^ (symmetric and asymmetric C−H stretching vibrations in aliphatic compounds) [45] exhibits similar effects, however, with lower variance in the absorbance density values.

In the range from 1800 to 800 cm^−1^ (the so-called fingerprint region), bands assigned to stretching and deformation vibrations of all wood components, more noteworthy changes in absorbance were recorded. The specimens treated at the lowest laser power (8%) displayed enhanced absorbance within the band of 1730 cm^−1^ (C=O stretching in unconjugated carbonyl groups), depending on the raster density used in engraving (Figure 8). At the two higher laser power values (12% and 16%), the absorbance of this band increased with increasing raster density (except for the highest density value, at which a moderate decrease was observed). These changes point to the changes in several functional groups in lignin and in hemicelluloses (carbonyls, aldehydes, ketones, and carboxylic acids) [46,47]. Increasing absorbance in the band assigned to the non-conjugated C=O groups indicates increasing amounts of acetyl and carboxyl groups in the lignin and in polysaccharides [48]. Reduction in this band could be, in our case, the result of hemicellulose deacetylation during the exposure of the wood surface structures [24]. 

Heat-induced processes are associated with the degradation of carbonyl groups in lignin and in hemicelluloses where the cleavage of bonds C=O affects the changes in the chromophores content. The chromophores are structures determining the wood surface colouring (this is also evident from the values related to the discolouration). 

The band at 1600 cm^−1^ (C=C stretching vibration conjugated with an aromatic ring in lignin) practically copied the performance of the band assigned to the carbonyl groups. The absorbance of the band around 1500 cm^−1^ (C=C stretching vibration and aromatic skeleton vibration) showed only small changes. This indicates decrease in amount of methoxyl groups confirming the reduction in the lignin content [49,50]. At higher temperatures, condensation reactions in lignin are possible [51,52]. Moderate changes, primarily dependent on the raster density were also recorded for the bands 1460 cm^−1^ (asymmetric CH_3_ bonding in methoxyl groups in lignin), 1370 cm^−1^ (symmetric and asymmetric CH_3_ bonding), 1320 cm^−1^ (C−O vibration in syringyl derivatives), and 1230 cm^−1^ (C−O stretching vibration in xylan and syringyl ring) assigned to the lignin and hemicelluloses [53,54,55,56]. The decrease in these bands has confirmed the supposition about bond-cleavage-caused degradation processes in lignin and the subsequent structural decomposition [57]. Contrarily to the hitherto observed trends, we observed a permanent decrease for the band 1030 cm^−1^ (C−O deformation vibrations in cellulose) (Figure 8). The absorbance on this band exhibited a permanent decrease indicating the degradation processes in the cellulose [58].

### 3.4. Discolouration of Engraved Surfaces

**Figure 9:**
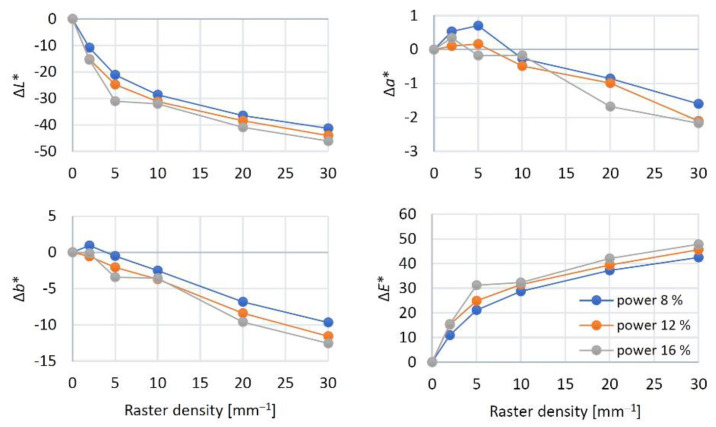
Raster density-dependent values of colour coordinates ΔL*, Δa*, Δb* and total colour difference ΔE* at varying CO_2_ laser power.

The interactions at the phase boundary wood–laser beam are complex. These interactions not only influence the changes in the wood surface structure and chemistry, but they also impact wood discolouration. The results of the two-way variance analysis confirmed the important effects of the two relevant laser parameters (laser power and raster density) on the colour variation in the engraved surfaces. The basic statistical characteristics of the colour coordinates L*, a*, and b* are listed in Table 3. The highest average lightness L* was measured in the referential specimens (L* = 68). With increasing raster density, at the given power values, the lightness decreased significantly down to the final values from 26.6 to 21.8, representing the lightness reduction by 61–68%. The colour coordinates a* and b* varied too. The differences in the individual colour coordinates ΔL*, Δa*, and Δb*, together with the total colour difference ΔE*, are in Figure 9.

The coordinate a* values were increasing with increasing raster density until 5 mm^−1^; after this raster value, a decrease by 25 to 33 % was recorded compared to the referential specimens (corresponding to the laser power). With increasing laser power and raster density, the coordinate a* exhibited a progressive loss in red saturation, because of a moderate shift towards an achromatic colour. The coordinate b* values decreased proportionally, over the entire raster range, at all the laser power values. The final decrease in this coordinate was from 41 to 63%.

**Figure 10:**
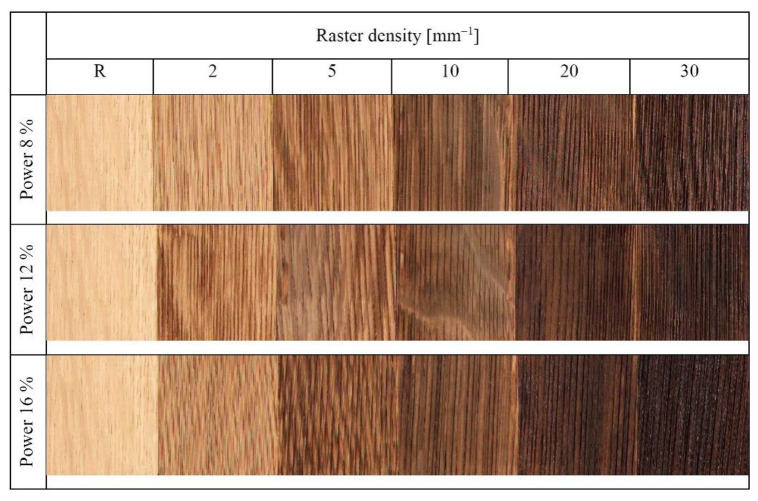
Oak wood surface treated with CO_2_ laser.

As for the discussed engraving parameters, the changes in colour coordinates were primarily a consequence of the varying the raster density. The effect of laser power has also been found statistically significant; however, in this case (Figure 9), the discolouration was less conspicuous. In addition, the colour-change effect of the laser power was partially over masked by the oak wood structure heterogeneity underlying the wood colour variability, as illustrated in Figure 10.

**Figure 11:**
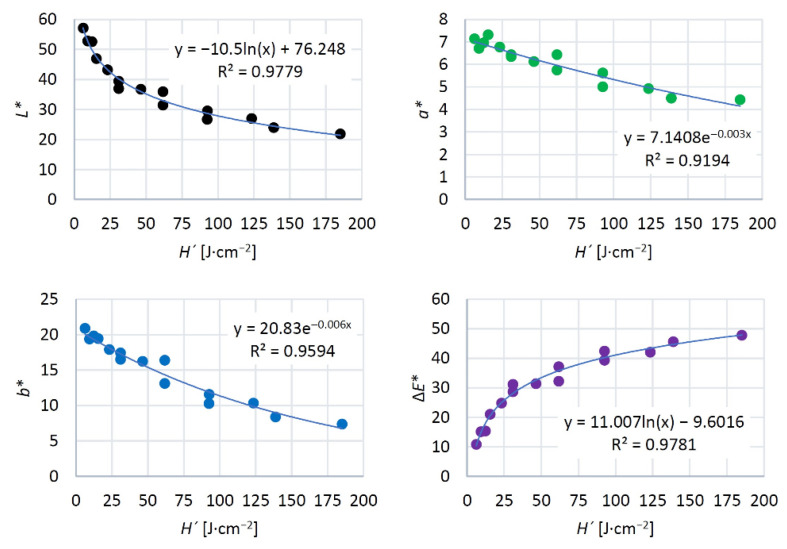
Colour coordinates and total colour difference depending on the irradiation dose generated by CO_2_ laser.

The impacts of the changes in the individual colour coordinates on the total colour change ΔE* are illustrated in Figure 9. Just at the minimum laser power (8%) and the minimum raster density (2 mm^−1^), the Δ*E* values calculated using Equation (7) ranged from 9 to 12, corresponding, according to the six-degree scale proposed by [33], to the degree five, expressing an easily visible change in relation to the original colour. With grading the laser power and raster density, the total colour difference values were far beyond 12, corresponding, in accordance with the reported scale, to the total new colour compared to the original. Qualitatively, these changes were like the ones reported by [10,11,16,20,24]. Quantitatively, our results were different, as we studied discolouration on different wood species and under varying laser parameters (head movement rate, focal distance and similar). This fact was an incentive for exploring the dependence of colour change on the total irradiation dose. The result was finding that all the radiation parameters (laser power, head movement rate, focal distance, and raster density) significantly influencing irradiation energy and wood colour could be substituted with a single variable H´, representing the total irradiation dose. The detected dependences of colour coordinates and total colour difference on irradiation dose are displayed in Figure 11. The results confirmed a strong dependence on the discussed colour coordinates on the total irradiation dose.

The quantification of the energy amount supplied onto the oak wood surface with a laser beam and the identification of irradiation-dose influence on discolouration provide conditions for targeted change in colour space in the relevant wood species treated with a CO_2_ laser. The detected dependence of colour changes on the total irradiation dose could enable engraving plastic patterns with a wider range of colour hues on the laser-treated surfaces for particular wood species, as has been discussed in [14].

### 3.5. Wetting and Surface Free Energy in Engraved Wood Surfaces

**Figure 12:**
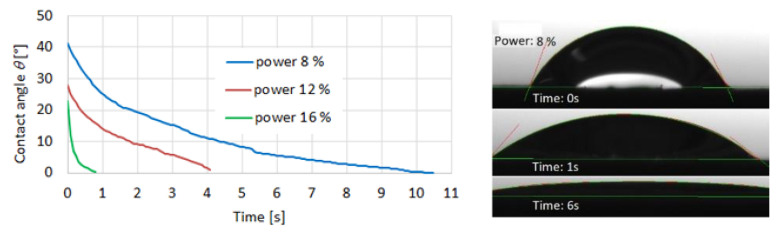
Contact angle variation during the process of laser-engraved oak wood surface wetting with water, up to the complete drop soaking into the substrate. Raster density of 20 mm^−1^.

**Figure 13:**
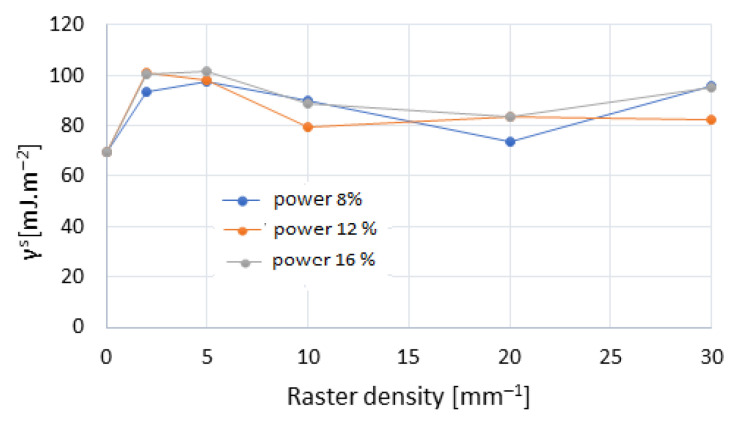
Surface free energy of oak wood surface, dependent on laser power and raster density.

Compared to the original oak wood surfaces, the engraved ones demonstrated much better wettability with both testing liquids. The results for oak wood wetted with water and diiodomethane are summarised in Table 3. Figure 12 illustrates the variability of the contact angles during the wetting process at a raster density of 20 mm^−1^, at all three laser power values. Each curve in the graph represents one particular characteristic measurement corresponding to the maximum contact angle at the moment of drop application onto the surface for the relevant treatment way. In many cases, the observed wetting was perfect, equally for water and for diiodomethane. This fact was also reflected in significantly higher values of surface free energy (Figure 13), ensuing from increased disperse and polar components (Table 4). These results are opposite to the results reported in [22], the authors of which observed enhanced hydrophobicity on pine wood surfaces treated with CO_2_ laser. The authors report pine wood surface temperature reaching up to 200 °C, without the surface carbonisation. The work [59] reports a significant increase in hydrophobicity of a heat-treated beech wood surface at 200 °C, which was reflected in contact angle values bigger than 90°. Worsened wetting of heat-treated modified beech wood was also observed by other authors [60,61].

The wetting values also differed from the results obtained for engraved beech wood surfaces by [11]. These different oak wood wetting values were caused by higher irradiation doses per unit area inducing more advanced destruction of lignin and cellulose, more frequent occurrence of carbonised layer, more pronounced roughness, and, consequently, enhanced porosity on the oak wood surface. On such surface, the liquid applied immediately soaked into the substrate.

The results of wetting and of surface free energy in oak wood surfaces treated with laser beam allow us to expect an appropriate adhesion of film-forming materials to wood. The pronounced roughness, however, may play a negative role in gluing such surfaces. Moreover, in the case of surfaces engraved at higher raster densities, it is necessary to consider an unstable carbonised layer (Figure 5), with weak adhesion, possible to peel off from the substrate easily.

## 4. Conclusions

The experimental results demonstrate that the studied laser engraving parameters (laser power and raster density) affected the wood mass loss, wood surface chemical structure, and morphology significantly, as well as the surface properties (colour and hydrophilicity) of the analysed oak wood. 

The energy amount supplied onto the wood surface under varying irradiation conditions was expressed through the total irradiation dose *H’.* This variable has been confirmed as an appropriate one for substituting all the irradiation parameters altogether (laser power, laser head movement rate, focal distance, and raster density). 

During the engraving, thin surface layers manifested immediate destruction and sublimation. The wood mass loss increased linearly with the irradiation dose. The wood mass destruction was not uniform over the whole surface. In the early wood with dominant early vessels, the trenches created by the laser beam were deeper than in the late wood, and this was reflected in the more pronounced roughness of the early wood.

The major impact on the roughness variability was detected for the raster density. With increasing raster density over the whole studied range the roughness parameters *Ra*, *Rz,* and *Rt* increased parallel with and similarly perpendicular to the grain. The more pronounced roughness as well as more distinct variability were recorded perpendicular to the grain course. The roughness parameter values *Ra* and *Rz* increased linearly with increasing irradiation dose.

The wood surface treatment with the laser induced wood discolouration. Already at the minimum laser power and the minimum raster density, evident discolouration could be declared. The next laser parameter rises caused a totally novel wood surface colour compared with the original one.

The discolouration was primarily due to heat-induced processes associated with the degradation of carbonyl groups in lignin and hemicelluloses in which the broken bonds C=O caused changes in the contents of chromophores determining the colour. 

A close dependence of discolouration on the irradiation dose was identified, regardless of the values of the individual irradiation parameters. This dependence can be declared as very important, because it serves a means for targeted discolouration of wood engraved with a CO_2_ laser.

The engraved surfaces exhibited significantly enhanced wettability with the standard polar and non-polar liquids, which was reflected in the higher surface free energy. The results concerning the wetting and the surface free energy values obtained for the laser-engraved oak wood surfaces allow us to suppose an appropriate spreading of film-forming material on wood surface, and, correspondingly, appropriate adhesion of film-forming materials to wood.

## Figures and Tables

**Table 1:** Irradiation dose per unit area, varying with laser power and raster density.

Power [%]		8	12	16
Power [W]		11	16.5	22
		Irradiation dose *H*′ [J∙cm^−2^]		
Raster density [mm^−1^]	2	6.17	9.26	12.34
	5	15.42	23.14	30.86
	10	30.84	46.28	61.71
	20	61.68	92.57	123.43
	30	92.52	138.85	185.15

**Table 2:** Basic statistical characteristics of roughness parameters, parallel with and perpendicular to the grain, in oak wood surfaces engraved under diverse laser power and raster density values (*x* represents the mean and *s* is the standard deviation).

Raster Density [mm^−1^]	Basic Statistical Characteristics	Roughness Parameters							
		Parallel to Grain				Perpendicular to Grain			
		*R*a	*R*z	*R*t	*RS*m	*R*a	*R*z	*R*t	*RS*m
		[µm]							
		Laser power 8% (11 W)							
0	*x*	5.15	38.18	88.59	1172.4	13.98	143.94	198.55	1937.7
	*s*	5.60	35.26	42.00	889.9	6.39	43.46	23.82	1081.1
2	*x*	20.20	106.08	163.25	3030.5	50.03	264.29	293.34	499.9
	*s*	8.72	47.15	51.41	1703.8	4.20	24.01	12.28	16.5
5	*x*	26.71	131.04	197.15	3733.2	48.97	328.43	400.99	589.6
	*s*	9.07	50.23	59.36	1796.1	7.46	51.44	54.56	233.7
10	*x*	25.70	150.89	260.14	2443.6	69.95	437.17	530.21	1721.0
	*s*	13.06	77.78	71.78	1506.8	12.66	55.72	52.56	605.6
20	*x*	35.09	202.19	303.87	2243.6	93.93	557.73	679.23	1870.2
	*s*	14.46	71.89	43.89	1315.2	19.34	76.24	46.09	681.7
30	*x*	35.53	194.48	287.90	2055.7	115.36	674.38	845.69	1714.0
	*s*	17.16	77.85	78.80	1216.5	25.16	124.54	124.22	524.3
		Laser power 12% (16.5 W)							
2	*x*	30.42	147.97	188.93	4155.59	55.79	273.97	308.85	506.59
	*s*	8.79	39.14	38.62	1975.23	4.41	30.54	20.16	15.51
5	*x*	28.88	133.51	184.58	3420.48	49.62	325.62	391.20	773.11
	*s*	8.39	37.83	41.68	908.59	8.70	44.87	30.31	519.89
10	*x*	28.61	159.14	262.52	2355.67	61.50	425.94	527.13	1659.11
	*s*	14.98	79.44	71.60	1135.97	19.53	85.64	46.08	684.26
20	*x*	30.45	179.38	277.84	2064.70	89.21	572.24	699.32	1830.35
	*s*	14.33	73.43	82.84	1494.56	22.07	88.62	70.45	841.47
30	*x*	36.88	212.60	305.65	1745.91	119.15	692.15	853.81	1658.83
	*s*	12.74	63.91	48.69	906.54	31.48	136.35	88.62	507.38
		Laser power 16% (22 W)							
2	*x*	17.96	93.10	137.54	2882.63	54.44	274.75	324.81	500.06
	*s*	9.37	45.12	45.81	1846.86	7.80	43.08	31.41	39.78
5	*x*	33.63	153.78	214.43	4172.09	58.37	378.12	464.81	429.51
	*s*	8.42	51.24	93.45	1788.46	8.81	70.56	42.47	292.40
10	*x*	30.19	174.69	271.51	2143.12	88.56	526.52	616.87	1841.38
	*s*	14.40	73.63	77.59	1302.42	16.66	74.71	56.94	605.22
20	*x*	28.30	167.79	263.61	1586.88	116.53	686.98	847.28	1660.33
	*s*	12.29	64.48	83.23	1020.57	24.94	109.32	49.51	459.69
30	*x*	37.50	223.32	343.89	1948.41	133.83	800.47	939.90	1779.32
	*s*	19.35	99.05	98.81	1188.91	20.76	95.43	64.99	436.58

**Table 3:** Basic statistical characteristics of colour coordinates for oak wood surfaces engraved with CO_2_ laser at different values of raster density and laser power (*x* represents the mean and *s* is the standard deviation).

Colour Coordinate	Basic Statistical Characteristics	Raster Density [mm^−1^]					
		0	2	5	10	20	30
		Power 8% (11 W)					
L*	*x*	67.97	57.10	46.88	39.35	31.43	26.65
	*s*	2.06	1.51	3.27	3.20	2.53	1.95
a*	*x*	6.61	7.13	7.31	6.34	5.75	5.00
	*s*	0.29	0.29	0.59	0.89	0.56	0.90
b*	*x*	19.96	20.92	19.46	17.45	13.14	10.29
	*s*	0.63	0.35	1.55	2.03	1.86	2.14
		Power 12% (16.5 W)					
L*	*x*	67.97	52.79	43.17	36.74	29.52	23.90
	*s*	2.06	1.52	3.31	2.02	2.25	2.37
a*	*x*	6.61	6.71	6.77	6.12	5.62	4.50
	*s*	0.29	0.49	0.85	0.84	0.62	1.22
b*	*x*	19.96	19.41	17.90	16.25	11.57	8.40
	*s*	0.63	0.98	1.46	1.13	1.55	2.17
		Power 16% (22 W)					
L*	*x*	67.97	52.57	36.92	35.91	27.00	21.85
	*s*	2.06	2.16	2.23	2.90	1.86	1.41
a*	*x*	6.61	6.95	6.43	6.43	4.93	4.43
	*s*	0.29	0.39	0.61	0.68	0.54	0.51
b*	*x*	19.96	19.83	16.55	16.39	10.36	7.41
	*s*	0.63	0.73	1.12	1.80	1.12	0.99

**Table 4:** Basic statistical characteristics for contact angles, disperse, and polar components of surface free energy, corresponding to varying raster density and to varying laser power (*x* represents the mean and *s* is the standard deviation).

Raster Density [mm^−1^]	Basic Statistical Characteristics	Contact Angles and Thermodynamics Characteristics					
		Water		Diiodomethane		Disperse and Polar Components	
		*θ*_0_	*θ* *_w_*	*θ*_0_	*θ* *_w_*	*γ* *_s_* *^d^*	*γ* *_s_* *^p^*
		[°]			[mJ·m^−2^]		
		Power 8% (11 W)					
0	*x*	71.6	34.0	44.0	43.0	38.0	31.6
	*s*	10.3	9.8	4.9	4.7	2.4	10.0
2	*x*	12.2	9.8	12.2	10.4	48.5	45.2
	*s*	16.4	13.1	16.4	14.1	3.4	7.8
5	*x*	5.2	4.5	5.2	6.0	49.2	48.3
	*s*	12.3	10.5	12.3	13.8	3.9	6.3
10	*x*	14.9	11.5	14.9	15.2	46.0	44.2
	*s*	16.6	13.8	16.6	18.1	5.48	8.4
20	*x*	30.7	28.1	30.7	36.5	39.9	34.0
	*s*	15.1	13.8	15.2	17.0	6.7	8.7
30	*x*	13.4	2.7	12.7	19.6	45.7	49.8
	*s*	40.1	7.0	13.4	18.7	5.9	1.1
		Power 12% (16.5 W)					
2	*x*	0.8	0.7	0.8	1.0	50.6	50.6
	*s*	3.5	3.0	3.5	4.2	0.5	1.5
5	*x*	4.4	3.6	4.4	4.7	49.3	48.7
	*s*	12.6	10.3	12.6	13.4	3.9	6.3
10	*x*	24.1	21.4	24.1	27.2	41.8	37.8
	*s*	22.2	20.2	22.2	25.4	8.9	12.5
20	*x*	19.1	18.1	19.1	23.6	43.6	39.9
	*s*	18.6	17.4	18.6	22.4	7.3	10.7
30	*x*	21.5	19.9	21.5	26.3	43.2	38.9
	*s*	17.0	15.4	16.9	20.0	6.3	9.4
		Power 16% (22 W)					
2	*x*	2.5	1.6	2.5	2.3	50.4	50.0
	*s*	7.4	4.9	7.4	6.8	1.2	2.7
5	*x*	0.0	0.0	0.0	0.0	50.8	50.9
	*s*	0.0	0.0	0.0	0.0	2.13	2.1
10	*x*	16.0	14.7	15.2	18.0	45.9	42.9
	*s*	40.4	37.9	16.0	19.2	6.0	9.0
20	*x*	20.0	18.6	20.0	24.7	44.0	39.8
	*s*	16.1	14.7	16.1	19.2	6.0	9.0
30	*x*	7.1	6.7	7.1	9.1	48.4	47.0
	*s*	12.5	11.8	12.5	15.7	4.4	7.0

## Data Availability

Not applicable.
